# Single Session Imaging of Cerebellum at 7 Tesla: Obtaining Structure and Function of Multiple Motor Subsystems in Individual Subjects

**DOI:** 10.1371/journal.pone.0134933

**Published:** 2015-08-10

**Authors:** Melissa A. Batson, Natalia Petridou, Dennis W. J. Klomp, Maarten A. Frens, Sebastiaan F. W. Neggers

**Affiliations:** 1 Brain Center Rudolf Magnus, Department of Psychiatry, University Medical Center Utrecht, Utrecht, The Netherlands; 2 Radiology Department, Imaging Division, University Medical Center Utrecht, Utrecht, The Netherlands; 3 Department of Neuroscience, Erasmus MC, Rotterdam, The Netherlands; 4 Erasmus University College, Rotterdam, The Netherlands; University of California, Merced, UNITED STATES

## Abstract

The recent increase in the use of high field MR systems is accompanied by a demand for acquisition techniques and coil systems that can take advantage of increased power and accuracy without being susceptible to increased noise. Physical location and anatomical complexity of targeted regions must be considered when attempting to image deeper structures with small nuclei and/or complex cytoarchitechtonics (i.e. small microvasculature and deep nuclei), such as the brainstem and the cerebellum (Cb). Once these obstacles are overcome, the concomitant increase in signal strength at higher field strength should allow for faster acquisition of MR images. Here we show that it is technically feasible to quickly and accurately detect blood oxygen level dependent (BOLD) signal changes and obtain anatomical images of Cb at high spatial resolutions in individual subjects at 7 Tesla in a single one-hour session. Images were obtained using two high-density multi-element surface coils (32 channels in total) placed beneath the head at the level of Cb, two channel transmission, and three-dimensional sensitivity encoded (3D, SENSE) acquisitions to investigate sensorimotor activations in Cb. Two classic sensorimotor tasks were used to detect Cb activations. BOLD signal changes during motor activity resulted in concentrated clusters of activity within the Cb lobules associated with each task, observed consistently and independently in each subject: Oculomotor vermis (VI/VII) and CrusI/II for pro- and anti-saccades; ipsilateral hemispheres IV-VI for finger tapping; and topographical separation of eye- and hand- activations in hemispheres VI and VIIb/VIII. Though fast temporal resolution was not attempted here, these functional patches of highly specific BOLD signal changes may reflect small-scale shunting of blood in the microvasculature of Cb. The observed improvements in acquisition time and signal detection are ideal for individualized investigations such as differentiation of functional zones prior to surgery.

## Introduction

### Cerebellar Function

**Fig 1:**
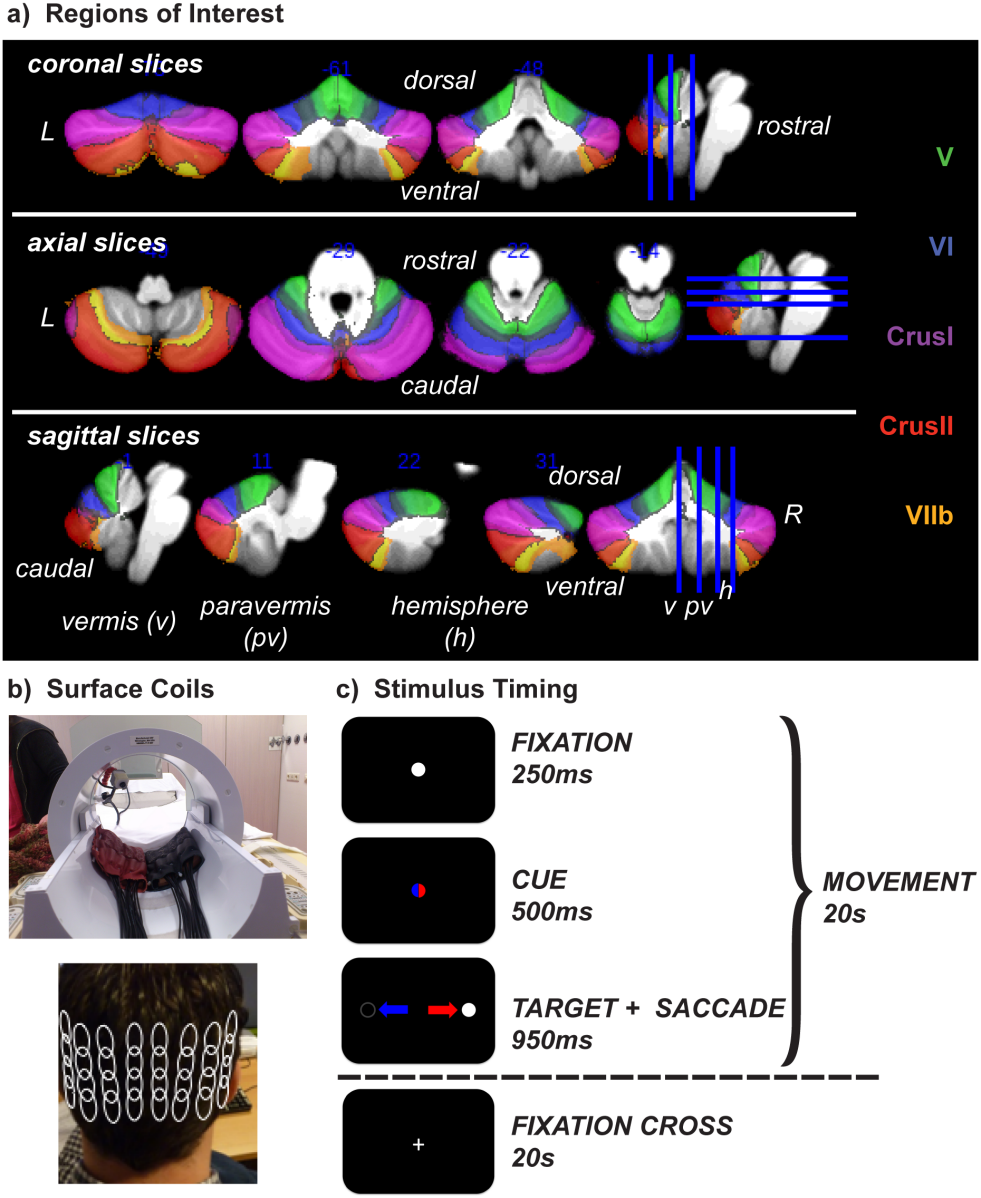
Experimental Design. **a)** Cb is divided into three main parts—vermis (medial Cb; will be denoted in the text with a “*v*”), paravermal (medio-lateral Cb) and hemispheres (lateral Cb; both of which will be denoted collectively in the text with an “*h*”)–which are then further divided into ten numbered lobules, arranged dorsoventrally I-X. Task-related activations were expected in five regions of interest: oculomotor vermis (*v*VI-VII) & *h*VIIb for the PA task, with possible cognition-related activation in lobules CrusI & CrusII (*h*VII), and in *h*V & *h*VIII for the FT task. These lobules are color-coded and overlaid on an average Cb from the SUIT toolbox [20] and listed, in matching colors, on the right. **b)** *top*–Two high-density surface coils with 16 elements each were used for signal reception (adapted from [21]), *bottom*–the coils were placed beneath Cb using the inion of the skull as a landmark and accurate placement was confirmed with a scout scan. **c)** The stimulus sequence was identical for both eye- and finger-movement tasks: In the pro-/anti-saccade (PA) task subjects made eye movements to locations which were either identical to (pro-saccade) or opposite from (anti-saccade) the location of a white target that appeared after a red or a blue central cue; color and movement direction pairings were counterbalanced across subjects. In the finger tapping (FT) task subjects moved their thumb at 2 Hz whenever the dots were moving. In each task the active period lasted for 20s and alternated with 20s of fixation for one full run of eight minutes.

The cerebellum (Cb) has a uniform architecture throughout and is divided into the cerebellar vermis (*v*) along the medial portion and cerebellar hemispheres (*h*) laterally with the paravermis (or intermediate zone) located between the two; these regions are divided into ten lobules, arranged dorsoventrally (Fig 1a). In general, Cb is able to update motor commands using on-line mechanisms to constantly adjust our participation with the external environment. For example, vision relies fundamentally on changing inputs in order to update visual information from the external environment; for this reason we constantly sample different locations in our visual world using fast eye movements (saccades). It is well known that the Cb is intimately involved in motor control [1–3] and that different regions of Cb react to or elicit distinct sensorimotor activity in different body parts (i.e. finger, arm, and eye movements, etc.) at both the cortical and nuclear layers [4–9]. A sensory/motor homunculus in Cb is mirrored in dorsal and ventral cortex [10] and somatotopic organization with specific regard to arm, hand, finger, and eye areas are all well defined in human and animal Cb—with hand and finger movements found topographically in dorsal motor areas (around ipsilateral *h*V); with a second, less strict, representation in *h*VIII, and eye movements in *v*II/III through VI ([9,11–18]; see [19] for review).

Different regions of Cb are implicated in higher order functions such as counting, timing, cognitive learning, and memory [2,19,22,23]. Many studies have implied that (lateral) Cb cortex is critical to cognitive or goal-directed neocortical processes involved in controlling volitional eye movements and/or appropriate suppression of reflexive eye movements, such as memory-guided saccades, anti-saccades, and saccade adaptation [24–28]. However, this viewpoint can be challenged, as attention and eye movements are an integral part of most studies on cognition [29–32]. Though it has been difficult to pinpoint the location(s) of different aspects of cognitive processes within Cb, relating the cognitive planning components of volitional movements (versus reflexive movements [33]) and/or motor learning are a good starting point.

While the general topography of functional zones in Cb is widely accepted, detailed location of function is not consistent across individuals [7,18] and this results in larger areas of activation in normalized space when assessing group analyses [34,35]. Enabling high-resolution structural *and* functional delineations for individualized assessment can be beneficial for many clinical procedures; e.g. for pre-surgical assessment of cerebellar infarcts related to vertebrobasilar stroke [36,37], tumor resection [38], or for individual lesion-symptom mapping [39,40]. Localizing these functional activations as quickly and succinctly as possible can further benefit this type of assessment.

### Imaging Cerebellum Is Difficult

The anatomical complexity and physical location of Cb creates many challenges to successful imaging of this structure. Differentiating the small-scale foliation of Cb cortex amongst local magnetic field inhomogeneities arising from neighboring air pockets (such as the ear canal), coil edge effects leading to loss of B1 power (which together can lead to significant ventral and lateral signal drop out), and tissue artifacts from neck muscle activity are important concerns when imaging these areas [15,34,41–43]. Minute structures and inhomogeneous location also make functional imaging of Cb more susceptible to physiological and movement artifacts [43–45], a particular confound when long acquisition times are required to obtain a significant contrast-to-noise ratio (CNR). With the advance of ultra-high field magnetic resonance (MR), high-resolution anatomical images and detailed localization of functional activations are possible. The increased spatial resolution enabled by high field MR systems has already fostered interest from the Cb research community, enabling feats such as visualizing granular and molecular layers of cerebellar cortex, creation of a probabilistic atlas for locating the deep cerebellar nuclei (DCN) and activity therein, relating activation of these nuclei to activations in Cb cortex, and visualization of somatosensory representations of the hand in lobules *h*V and *h*VIII in individual subjects [18,46–49]. These studies exemplify the ability to target deeper structures with small nuclei and/or complex cytoarchitechtonics (i.e. small microvasculature and/or deep nuclei) at high field strengths. Despite these advances, high-field Cb studies typically focus on either structure *or* function of a single system (e.g. somatosensory finger topography). Thus the ability to clearly differentiate the entire region of interest (ROI; in our case, both Cb cortex and DCN) structurally and acquire functional information about multiple systems within a single session remains unseen.

### Combining Techniques to Tackle the Challenge

These concerns demonstrate the need for acquisition methods and coil systems that can take advantage of the increased signal at high field strengths whilst controlling for increases in signal dropout and artifacts, such as those arising from local B0 (constant, homogenous magnetic field) or B1 (applied radio frequency (RF) energy field) inhomogeneities, especially at the level of Cb. Recent developments in acquisition schemes, such as the use of multiple receiving coils and accelerated (3D) parallel imaging employing sensitivity encoding (SENSE), allow for increases in the spatial resolution of images without increasing acquisition time. These acquisition schemes are also able to maintain a high signal-to-noise ratio (SNR) and bolster image attributes such as blood oxygenation level-dependent (BOLD) signal specificity and temporal SNR (tSNR) [50–52]. A second advance which successfully combats inhomogeneity problems is the use of multiple RF amplifiers to steer constructive and destructive B1 field patterns, subsequently increasing B1 homogeneity and allowing uniform excitation of the volume and further reducing signal dropout [53,54].

Despite advances in acquisition techniques, spatial restrictions of scanner hardware remain as a technical barrier that must be overcome to facilitate many experimental paradigms. For example, when imaging the whole brain, the solid head coils required to provide a uniform B1 field whilst receiving signals from the entire head limit the ability to easily present visual stimuli to subjects and are sometimes too small for subjects with large head circumference. However, concentrating coil density over a posterior ROI in the brain can clear up space for presentation of visual stimuli and/or subject comfort whilst increasing the local homogeneity of the B1 field around the ROI [18,21,55]. Moreover, studies using a high-density multi-element surface coil to detect BOLD signal changes at ultra-high spatial resolution benefit from increased SNR and CNR, indirectly decreasing acquisition time.

Here we aimed to clearly reveal subject-specific task-related activity in Cb associated with two well-documented motor subsystems, eye-movements and finger-movements. The end goal was to confirm that high-density surface coils maintain improved and uniform tSNR and BOLD contrast over the entire Cb in healthy individuals. The combinatorial methodology used in this study also overcomes the aforementioned challenges of imaging deeper structures, shortening acquisition times to increase experimental efficiency and subject comfort, and relieving spatial constraints within the transmit coil. The acquisition scheme utilizes two 16-channel surface coils of [21] integrated in a volume transmit coil (Nova, USA) powered by two RF amplifiers, which were shimmed separately, and a 7 Tesla (7T) scanner. Images were acquired with 3D-parallel imaging using SENSE and B0 shimming. A task including both pro-saccades and anti-saccades (PA task; inducing both reflexive and volitional saccades, respectively) was used to confirm eye-movement-dependent activations in oculomotor vermis (OMV, *v*VIc and *v*VII) and DCN [9,32,56–58]. Additionally, the role of lateral Cb cortex in goal-directed eye-movement planning was also probed with this task, with expected activations in *h*VI and/or CrusI/II relating to volitional saccades, visual attention, and/or saccadic errors from anti-saccades [25,59,60]. A finger-tapping (FT) task was used to confirm activations in ipsilateral dorsal and ventral (sensory-) motor hand areas of Cb cortex [11,15,17,18], *h*IV-V and *h*VIII, which should not overlap with the oculomotor system.

## Materials and Methods

This study was approved by the Medical Ethics Committee of the University Medical Center Utrecht (METC approval no. 07-235/C) and all subjects gave written informed consent prior to participation.

### Subjects

Seven right-handed subjects with normal or corrected-to-normal vision participated in the experiment (four male). The average age of subjects was 31. No subject had a history of mental or neurological illness; all were screened for implanted metal objects before entering the fMRI experiment.

### Acquisition

Scanning was performed on a Phillips 7T scanner (Phillips, Best, NL) with a gradient strength of 40 mT/m and a slew rate of 200 T/m/s, using two dedicated 16-channel surface receiving coils (MR Coils BV, Drunen, the Netherlands; for a total of 32 channels; see Fig 1b and [21] for more details on coil arrangement and design, respectively) with a volume transmit coil (Nova Medical, MA, USA) and dual transmission for excitation. Cb was located using the inion as a landmark, and placed on the center of the surface coils. Before shimming, accuracy of placement was confirmed with a scout scan and subjects were repositioned if Cb was not within the field of view (FOV) of the surface coils. (For an example of data of one subject acquired when the Cb was below the FOV of the coils, see S1 Fig) RF transmit phases were adjusted separately to homogenize the B1 field around Cb and the B0 field was shimmed separately on the FOV using pre-defined shim tools built in house for both procedures [61]; these shimming parameters were then applied to all subsequent acquisitions, including the coil sensitivity profile acquisition. Functional and structural scans were obtained with a 3D acquisition protocol (see [51,52] for examples) using SENSE [50]. FMRI data were acquired using a segmented 3D-echo planar imaging (EPI) sequence with the following parameters: effective *TR/TE* = 42/25 ms; FOV (right-left, foot-head, anterior-posterior) = 140 x 160 x 50 mm^3^; flip angle (FA) = 20°; with an EPI factor of 29 and 40 coronal slices; voxel size = 1.25 mm isotropic (BW 1355.1 Hz); SENSE factors *R* = 2.3 (Right-Left) and *R* = 1.5 (Anterior-Posterior). The echo-train duration was 28 ms, and total acquisition time per volume was 2940 ms, yielding 164 acquisitions for one eight-minute run. Anatomical T1-weighted (T1w) MPRAGE parameters were: *TR*/*TE* = 8.0/3.1 ms; FOV = 140 x 160 x 50 mm^3^; FA = 10°; voxel size = 0.63 mm isotropic; *R* = 1 in all directions; total acquisition time was 5’52”. T2-weighted (T2W) scan parameters were: *TR/TE* = 3182.5/2.6ms; FOV = 180 x 180 x 58 mm^3^; FA = 50°; EPI factor = 13 and 10 coronal slices; voxel size: 0.28 x 0.28 x 4 mm with a 2mm gap; total acquisition time was 4’46”. Anatomical T2*-weighted (T2*w) scan parameters were: *TR/TE* = 50.91/27 ms; FOV = 152 x 152 x 35 mm^3^; FA = 24°; with an EPI factor of 13 and 70 coronal slices; voxel size = 0.5 x 0.5 x 0.5 mm^3^; total acquisition time was 46.2 seconds. T2W and T2*w scans were only collected from the last two subjects to investigate DCN and vasculature. Total time in the scanning room, including shimming, was 40 to 55 minutes.

### Task-Related Changes in BOLD Sensitivity

Task-related changes in BOLD sensitivity and specificity were assessed in seven subjects using a block-based design of two motor tasks well known to activate specific areas of the cerebellum (ROIs, Fig 1a): *v*VI and *v*VII for pro-saccades versus anti-saccades (PA), and *h*V and *h*VIII for finger tapping (FT). See Fig 1a for structural locations of ROIs and 1c for a schematic of stimulus timing. The same visual stimuli were used for both tasks. In the active block of both tasks, a colored central cue was presented at fixation for 500 ms, and at the offset of the cue a white target appeared on the left or right at 3 or 10 degrees of visual angle (DOV) from fixation for 950 ms; a white central cue guided the eyes back to the center of the screen for 250 ms. Both cue and target subtended approximately 3 DOV. Each active block lasted 20 seconds, and active blocks were alternated with rest blocks (20 seconds of fixation) for one full run lasting eight (8) minutes. For the PA task, subjects were instructed make a pro-saccade (towards the target) when the cue is red and to make an anti-saccade (away from the target, to the un-cued mirror location) when the cue is blue; color cues were counterbalanced over subjects. For further task details see [62]. For the FT task, subjects were instructed to move their thumb at 2 Hz whenever they saw dots moving on the screen, and to rest during the fixation period. Visual stimuli were presented on a screen above the transmit coil. Images were reflected from the screen via an adjustable intermediate mirror and viewed through prism goggles, which re-directed the image from this mirror towards the eyes.

### Spatial Preprocessing

All image processing and statistical modeling was done with SPM8 (Wellcome Trust Center for Neuroimaging, London, UK) on MATLAB 7.12.0 (Mathworks, Natick, MA, USA). Functional images were realigned and resliced at their original voxel size and all structural and anatomical images were coregistered to the mean functional image. Using the full-head scout scan as a guide, the anterior commissure (AC) was moved to [0 0 0] xyz coordinate and the head was rotated to approximate a common atlas position by eye, these transformations were applied to the header of all images to approximate Montreal Neurological Institute (MNI)/Talairach space. Cb was isolated from the T1w-image using the SUIT toolbox [20]. Note that though the AC of each scan was centered, no normalization was applied to any scans for individual analyses. Functional images were smoothed at 2mm FWHM and nuisance regressors were extracted from the time series on a per-subject basis using 3 x 3 x 3 mm^3^ samples from white matter in the left and the right hemisphere and from cerebral spinal fluid (CSF) in ventricle IV, the superior cerebellar cistern and the cisterna magna. The parameters from the SUIT isolation were later used to normalize functional acquisitions to common SUIT space on a per-subject basis to confirm individual results in a common space and for analyses at the group level (see *Spatial post-processing and group statistics*, below).

### Statistical Analyses

#### Evaluation of signal quality and stability

Signal quality of these surface coils has been previously defined for visual cortex [21,55] showing boosted tSNR close to the coil, yet with a limited depth of view, which is required to cover the entire Cb. While the performance assessment in those studies was obtained with a single 16 channel surface coil, we required deeper penetration in order to reach Cb and therefore used two sets of 16 channel surface coils and two transmission amplifiers. To confirm sufficient SNR over the Cb with this arrangement, tSNR was computed from one run of PA acquisitions in a single subject (S05), after motion correction and removal of the task-induced signal, by calculating the residual variance of the remaining signal and normalizing the average signal intensity by this variance (over time), for each voxel in the brain. This subject also completed both tasks in a standard 32-channel full head coil (Nova Scientific, USA) with identical scan parameters and analyses (see below), and the resulting activation maps were compared to the surface coil acquisition; see S1 Methods for details.

#### Changes in brain activation

A GLM contrasting 20s task against 20s rest was created using the block design from each experimental run convolved with the canonical hemodynamic response function (HRF), and a high-pass filter of 80s was applied to each timeseries; this model also included the aforementioned WM and CSF nuisance regressors. Timeseries from all voxels within the imaged volume were included when estimating the model. The resulting T-maps from this active versus rest contrast were overlaid on the T1w images using MRIcron [63]. Voxels with significance above *p* < 0.05 (voxel-based family-wise error (FWE) false discovery rate (FDR) corrected, automatically calculated with SPM interface, further referred to as ‘SPMT’) frequently occurred in extremely small clusters that are difficult to make out at any scale without significant prior knowledge of Cb anatomy. This default FDR is a very strict correction for individual analyses and does not weigh the individual variations in noise. Therefore a cluster-based FDR correction utilizing the intercepts of the noise Gaussian distribution with the positive and negative gamma signal was also applied, specified for each subject individually (adaptive thresholds, ‘AT’ [38]). The AT maps were then thresholded, removing voxels below the AT and/or not surviving FDR cluster thresholds. Voxel clusters surviving SPMT therefore needed to exceed the minimum size of corresponding AT clusters to survive this thresholding method. Individual T-values in each figure therefore range from cluster-based AT FDR to voxel-based SPMT FDR values representing *p* < 0.05 in both instances.

#### Spatial post-processing and group statistics

To assess the stability of the results across the sample size of this experiment, statistical maps from each subject were created from the SUIT-normalized and smoothed functional images masked inclusively for voxels within the standard SUIT Cb mask. All resulting maps were overlaid on the SUIT atlas to investigate the validity of the individual activations in common (MNI) space. These maps were then entered into two simple second level analyses (Paired t-Test and multiple regression only) in SPM to evaluate activations at the group level. No additional second level analyses were run, as the goal of this study was to investigate activations at the single-subject level.

## Results

The data of one subject were acquired when the Cb was below the FOV of the coils and these data are not displayed in the figures of the main text; see S2 Fig for complementary panels of structural and functional images from this subject.

### Task-Related Signal Changes

**Fig 2:**
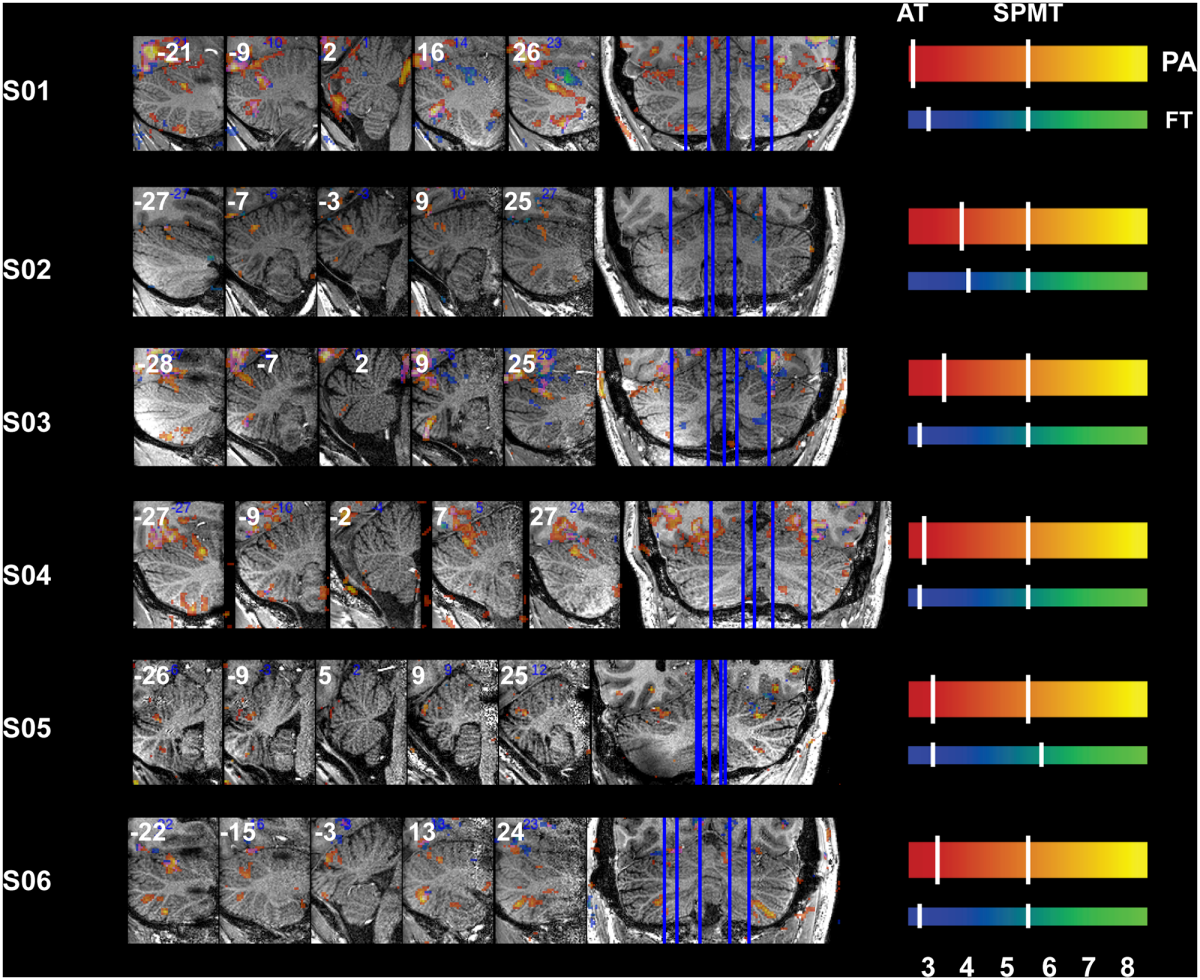
Functional Accuracy of Pro-Anti Activations in Cb, Oculomotor Vermis and Paravermis VI. Activity in OMV (medial posterior Cb, layers *v*VIc and *v*VII, central panel) and paravermis VI and CrusII/VIIa during the PA task is displayed on sagittal slices (vertical lines dissecting rightmost coronal view). Ventral OMV is active in all subjects besides S05 during this task while activity is also present in dorsal OMV for all subjects besides S03 and S04; slices are through Cb vermis and paravermis only. Slice locations (sagittal non-normalized MNI space, x-plane) are displayed at the top of each panel. Refer to Fig 1a for a guide to anatomical lobule definitions.

**Fig 3:**
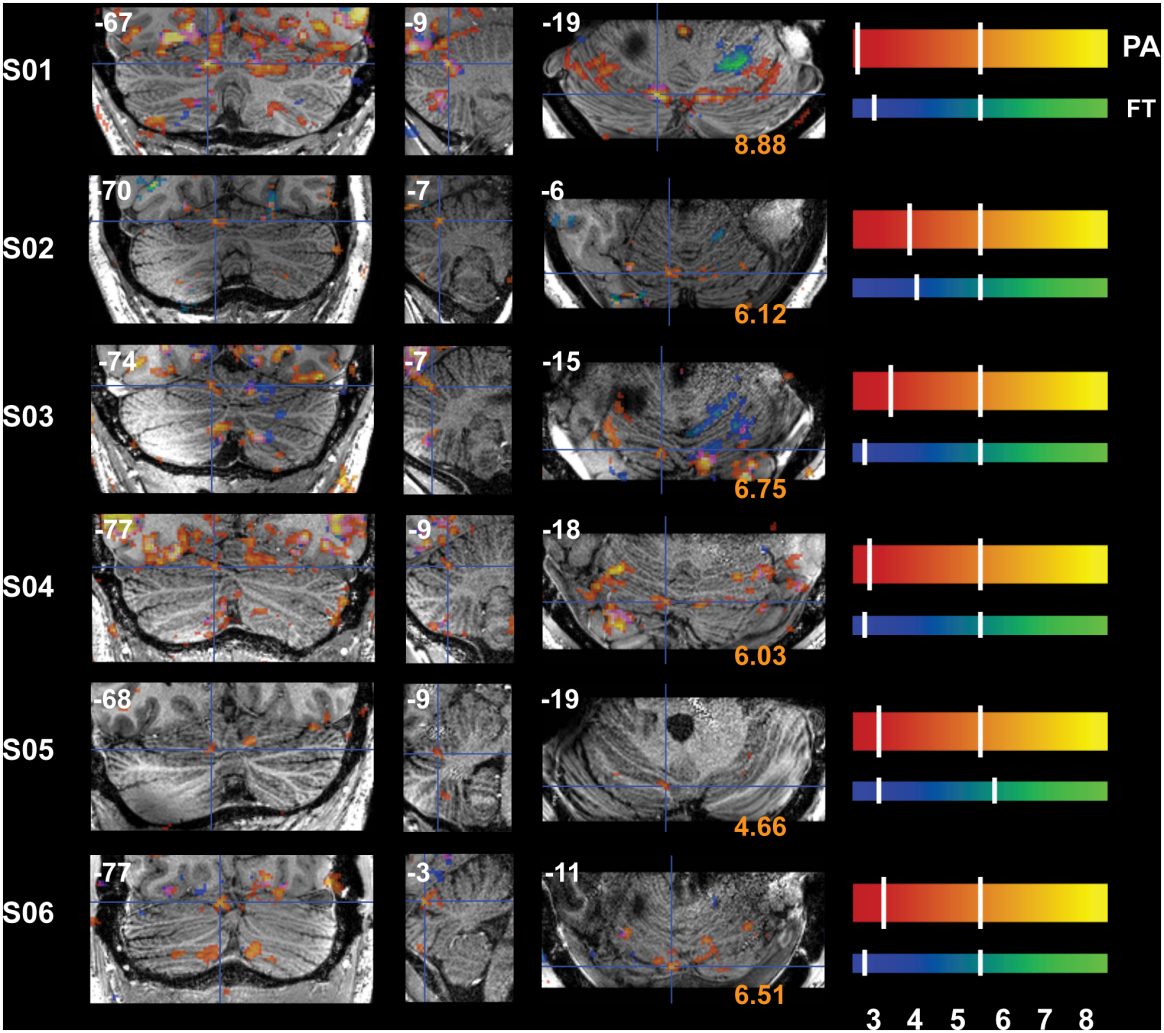
Functional Accuracy of Pro-Anti Activations in Cb, Bilateral VI. Bilateral *h*VI is activated during PA; crosshairs denote an active cluster in left paravermis VI and distinct clusters can be seen aligned along lobule VI in the axial (rightmost) panels. Slice locations (in non-normalized MNI space) are displayed at the top of each panel and T-values at the crosshairs are displayed at the bottom of the axial panels. Refer to Fig 1a for a guide to anatomical lobule definitions.

**Fig 4:**
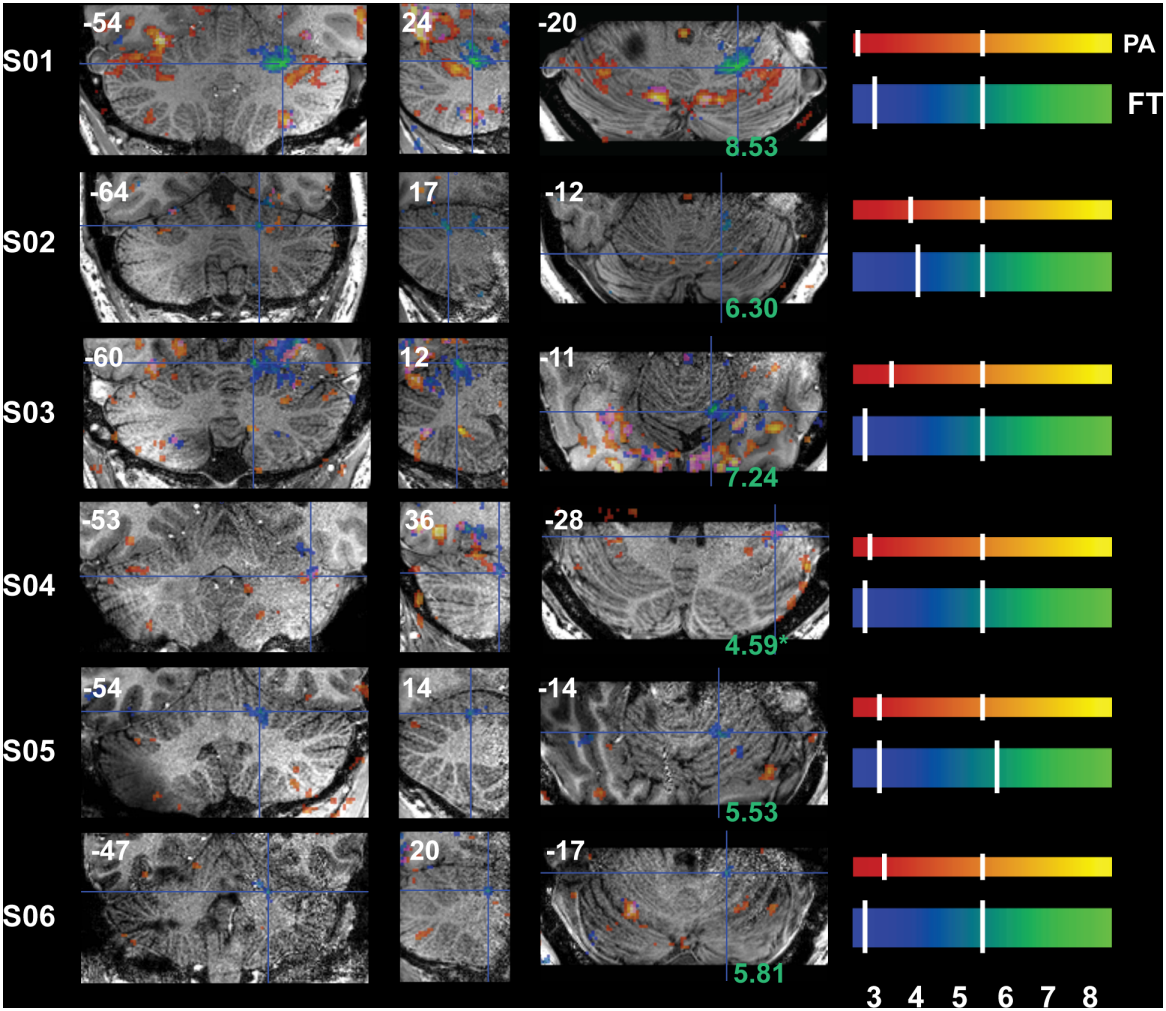
Functional Accuracy of Thumb Tapping Activations in Cb, Ipsilateral IV-VI. Ipsilateral *h*IV*-*VI is activated during the FT task; crosshairs denote an active cluster in or adjacent to right *h*V (S04 –*h*VI-VII). Activations for both tasks are shown in all images, with PA activity denoted by red-yellow and FT activity by blue-green color bars. Note the large structural and functional (with regard to both location and strength) variability between subjects; maximum FT-clusters can be located anywhere between the IV-V border (S03, S05 and S06) to the V-VI border (S01, S02, S04). Slice locations (in non-normalized MNI space) are displayed at the top of each panel and T-values at the crosshairs are displayed at the bottom of the axial (rightmost) panels. Refer to Fig 1a for a guide to anatomical lobule definitions.

**Fig 5:**
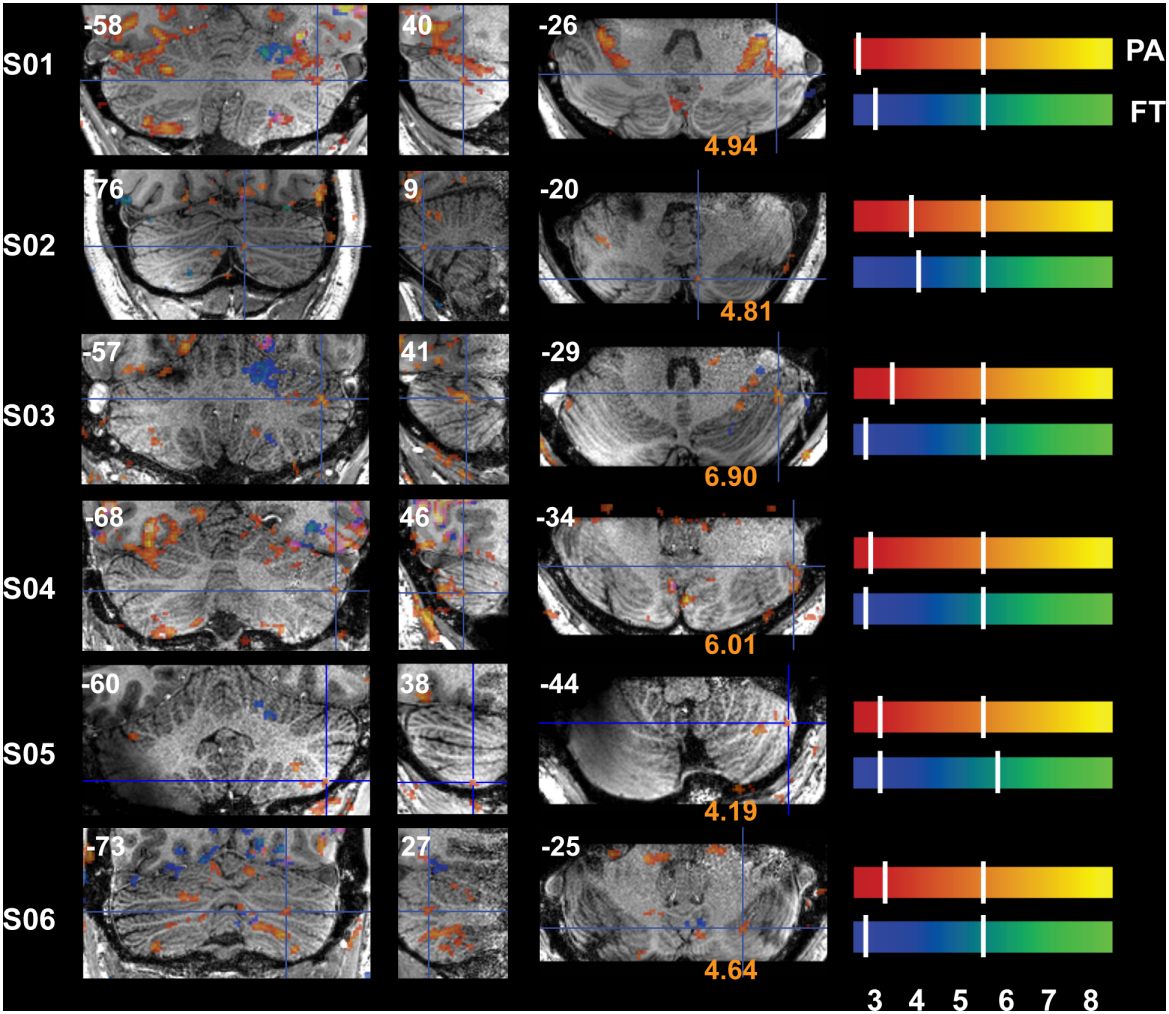
Functional Delineation of Task-Related Activity, Pro-Anti Activations in CrusI and CrusII. PA-related activity is also seen in *h*CrusI and/or *h*CrusII (crosshairs); presumably due to the cognitive component required to plan and execute a volitional anti-saccades. Clusters occur in either dorsal CrusI (S01, S03 and S06) or medioventral CrusII (S02, S04 and S05). Slice locations (in non-normalized MNI space) are displayed at the top of each panel and T-values at the crosshairs are displayed at the bottom of the axial (rightmost) panels. Refer to Fig 1a for a guide to anatomical lobule definitions.

**Fig 6:**
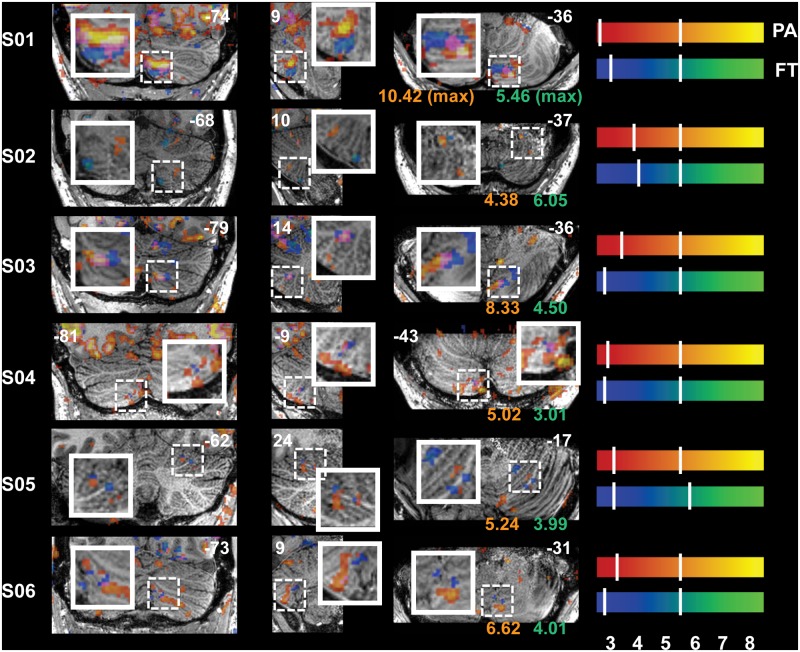
Functional Delineation of Task-Related Activity, Separation of Eye and Finger Movements in Ventrolateral Cb. Example of the specificity of localized activity resulting from the two motor systems (in addition to the obvious distinctions visible in Figs 2–5): differentiation of FT-related *h*VIII activity from PA-related *h*VIIb activity is displayed for each subject (zoomed-in area denoted by a dotted box); activations from separate tasks do not overlap. Separable activations are located more anteriorly in some subjects (S02, S05 and S06, axial slices) and the proximity and arrangement of the clusters also varies between subjects. Slice locations (in non-normalized MNI space) are displayed at the top of each panel and maximum T-values from within the zoomed areas are displayed at the bottom of the axial (rightmost) panels, color-coded by task—PA in red-orange and FT in blue-green. Refer to Fig 1a for a guide to anatomical lobule definitions.

**Fig 7:**
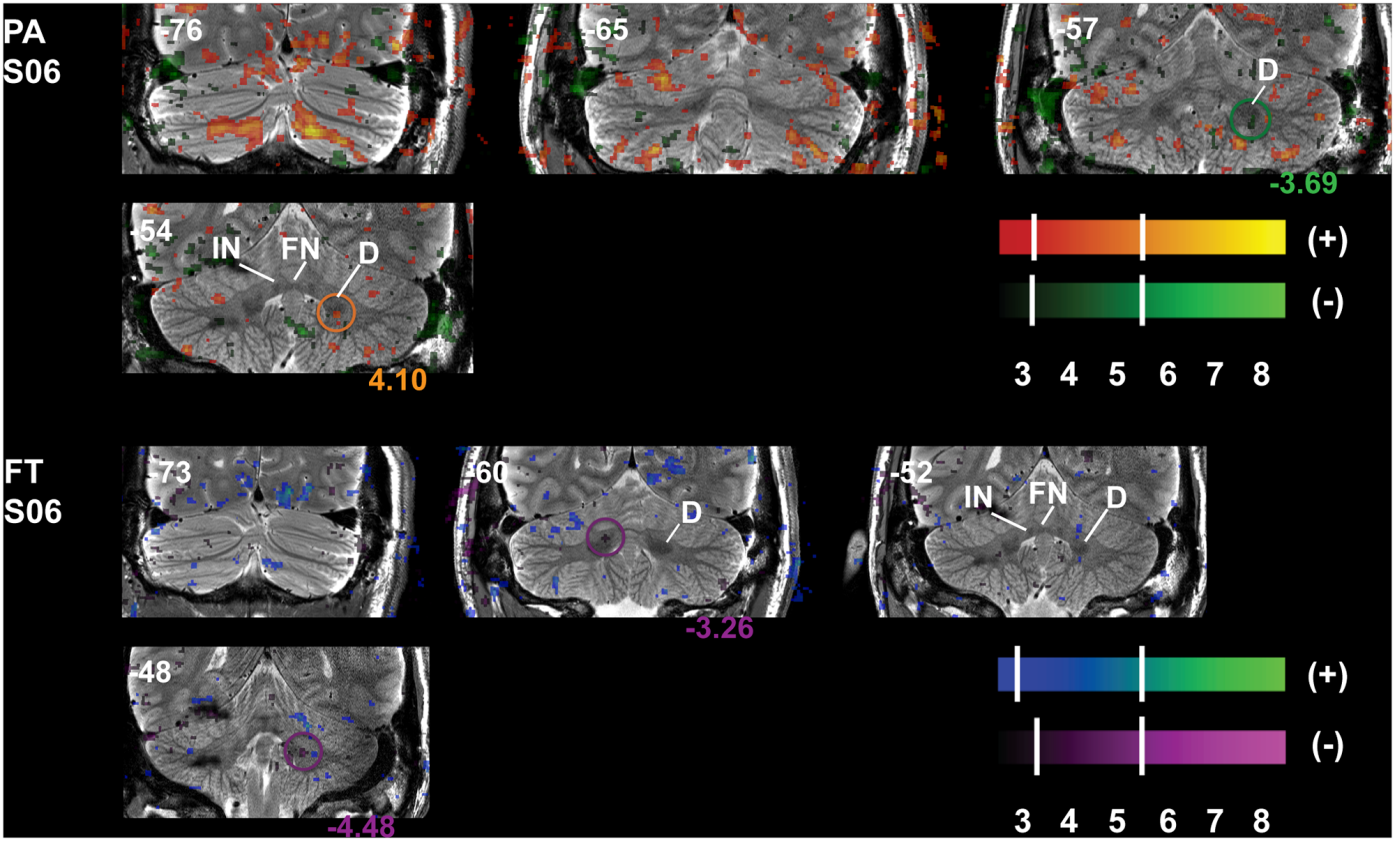
Concomitant Correlation of Task-Related Cb Cortex Activity and Anticorrelation of DCN Activity. The detail of the T2W scan allows visualization of DCN revealing increases (+) and decreases (-) in task-related functional activity in Cb cortex and within the dentate (respectively) of a single subject. Correlation of task-related activity (+) in the Cb cortex is concomitant with anticorrelation of activity (-) in the dentate and that cognitive depression (PA, green at -57) is located more caudal in the dentate than (the stronger) motor depression (FT, purple at -48). Slice locations (in non-normalized MNI space) are displayed at the top of each panel and maximum T-values from clusters within the circles are displayed at the bottom of the panels; coloring matching the respective color bar. Refer to Fig 1a for a guide to anatomical lobule definitions. FN—fastigial nucleus, IN—interpositus nucleus, D—dentate nucleus.

Reliable signal changes were observed within Cb when contrasting active versus rest periods for both tasks in all subjects. Figs 2–6 show the specificity of task-related Cb activation in each subject in non-normalized space as overlays on individual T1w structural scans. In all images, overlays are cluster-thresholded AT maps and the color bar(s) denote a range of T-values encompassing the *p* < 0.05 thresholds for the two types of FDR thresholding analyses run: from *cluster-based AT FDR values* (lower-value hash mark on each color bar) to *voxel-based family-wise error (FWE) SPMT FDR values* (higher-value hash mark on each color bar). In Figs 2–6, subject-specific data are displayed in the panels beside the subject ID and activations for both tasks are shown in all panels: PA activation is displayed in red-to-yellow colors while FT activation is displayed in blue-to-green colors (each representing low-to-high significance, respectively). Hash marks through the color bars indicate the lower (cluster-based) and upper (voxel-based) FDR thresholds. See S1 Table for a list of AT and SPMT values for each task for each subject. In addition, the maximum T-value at the crosshairs or within the circles in Figs 3–7 is displayed on the axial slice, color-matched to the color bar representing each task.

Fig 2 displays sagittal slices through the paravermis and OMV (*v*VI*-*VII) and Table 1 lists the maximum T-value per cross-section for each subject. Fig 3 displays crosshairs in paravermis (left *h*VI) where activity was indeed observed in each subject during PA eye movements. Note that activations were confined to the grey matter and that the pinpoint PA-related activity is located in clusters bilaterally along lobule VI in the axial plane (rightmost panels: vermis, paravermis, and hemispheres), seen as bright yellow activations in line with the curvature of the lobule marked by crosshairs in the axial panels of Fig 3. Aside from the differences in sagittal (left-right) locations dissecting the PA-related clusters, the PA-clusters are themselves located at slightly different axial (foot-head) locations. Ventral OMV is active in all subjects besides S05 during this task while activity is also present in dorsal OMV for all subjects besides S3 and S04 (Fig 2).

**Table 1:** Oculomotor Vermis Coordinates and Maximum T Values.

S01	x = -21	x = -9	x = 2	x = 16	x = 26
	7.4614	8.8765	3.0502	7.1134	9.4295
S02	x = -27	x = -7	x = -3	x = 9	x = 25
	6.137	6.1186	6.1169	5.0553	4.9984
S03	x = -28	x = -7	x = 3	x = 9	x = 25
	5.8972	6.7514	4.1853	7.9593	6.5249
S04	x = -27	x = -9	x = -2	x = 7	x = 27
	9.3242	6.0276	4.9292	7.5602	7.8195
S05	x = -26	x = -9	x = 5	x = 9	x = 25
	5.0573	4.6583	3.9448	5.4824	5.5194
S06	x = -22	x = -15	x = -3	x = 13	x = 24
	7.8266	5.2332	6.5102	5.9104	4.6537
S07	x = -8	x = -5	x = 6	x = 30	x = 31
	NA	3.0536	3.5386	3.5286	2.9817

Crosshairs in Fig 4 denote an active cluster in right *h*V where finger-movement related activity was also observed as expected and reliably in each subject. Also note the restriction of FT-related activity to a small number of folia within ipsilateral (R) *h*V-VI (Fig 4). Although activity was observed in or around the expected lobule of each subject, the location of peak activity varied between subjects. For instance, the PA-clusters that spill over from ventral V to dorsal VI in S01 lie more dorsally in layer V in S03 and S04 (sagittal sections, Fig 2), and maximum Cb FT-clusters can be located anywhere between the IV-V border (S03, S05 and S06) to the V-VI border (S01, S02, S04; Fig 4)

In addition to the expected motor-related activity mentioned above, lateral Cb was found to be active during the PA task for most subjects (particularly prominent in S01, S03, S04 and S06; Fig 5), presumably representing the cognitive load of planning volitional anti-saccades and/or visual attention. Clusters of activity were observed in *h*CrusI and/or *h*CrusII as well as *h*VIIb during the PA task (Figs 5 and 6, respectively). Note that the most active clusters occur in either dorsal CrusI (S01, S03 and S06) or medioventral CrusII (S02, S04 and S05) and that activity in lateral Cb was also significant with the head coil (S05, S1 Fig) though FDR clusters are smaller and more localized with the surface coils. Eye-movement and finger-movement activations were also consistently non-overlapping in ventrolateral Cb. It can be seen throughout Fig 6 that FT activity in ipsilateral *h*VIII, the secondary motor lobule, is consistently differentiated from PA activity in bilateral *h*VIIb with the exception of S04 where this differentiation was only visible in contralateral Cb, and S05 where ventral FT activity was extremely low overall and separation in dorsolateral VI is displayed instead (this dorsal separation is also visible in Fig 4 for other subjects). Once again, though differentiation is consistent across subjects, the relative strength and positions of the activations are not. For example, the separable activations in Fig 6 are located more anteriorly in half of the subjects (S02, S05 and S06, axial slices) and more posteriorly in the other half; and the proximity and arrangement of the clusters are closer together in some subjects than in others.

BOLD signal changes were also detectable in the DCN. The inhibitory nature of Purkinje cell (PC) afferents from Cb cortex to ipsilateral dentate nucleus (D) mean that activations in the PC layer (middle Cb cortex layer) should result in deactivation of D. Fig 7 displays the dentate (D) and surrounding DCN identified on the T2W structural scan of a single subject. Activations are shown as voxels from the original (un-thresholded) SPM T-maps since no D activations survived the AT cluster-thresholding; hash marks denote the AT (lower value) and SPMT (upper value) for positive (top color bar) and negative (bottom color bar) activations and deactivations (anticorrelations), respectively. It can be seen in Fig 7 that activity in D is anticorrelated with activity for both tasks: when Cb cortex is active, D is not as seen during PA at -57 mm (circled in green) and during FT at -48 mm (circled in purple). The (cognitive) goal-directed eye movement connections (PA) are located more caudally than finger-movement motor connections (-57 versus -48mm, respectively), yet both occur in ventral D. It should be noted, however, that positive activity is also seen in more rostral D during PA and that no activity was observed in interposed or fastigial nuclei of this subject.

### Structural Detail, Signal Quality and Stability

The conspicuity of structural detail in the T1w scan is apparent, with remarkable definition of the folia, especially around the edges of the Cb (Figs 2–7) where inhomogeneity was most likely. Both SNR and tSNR have previously been validated in visual cortex for the surface coils [21,55]. S1 Fig shows the advantage of the high-density surface coils in Cb functional imaging—PA-related activity in lateral Cb was detected using both the head and surface coils (see also S05 in Fig 5 and S3 Fig). With regard to tSNR throughout Cb, signal loss further than 5cm from the surface coils (anterior cerebellum, towards the center of the brain) was indeed an issue, but a minimal one. T2*w and T2W images were collected from the last subjects to visualize differences in local vasculature and anatomy. S3 Fig displays functional activations from the PA run of S05 overlaid on T1w images (‘Funct’) and aligned with separate panels displaying tSNR and T2*w structural images for six defined locations. Task-related activity was neither directly related to local tSNR nor to the proximity of large vessels, as revealed by comparing the location of functional activity with the tSNR and blood vessels/veins at the same location (crosshairs for each defined location are aligned for all images types). As described in the previous section, the T2W anatomical image was used to visualize the DCN with great clarity, particularly D, in S06 (Fig 7), and functional activations and deactivations were observed within these deep nuclei.

### Inter-Subject Variability and Statistical Post-Processing

**Fig 8:**
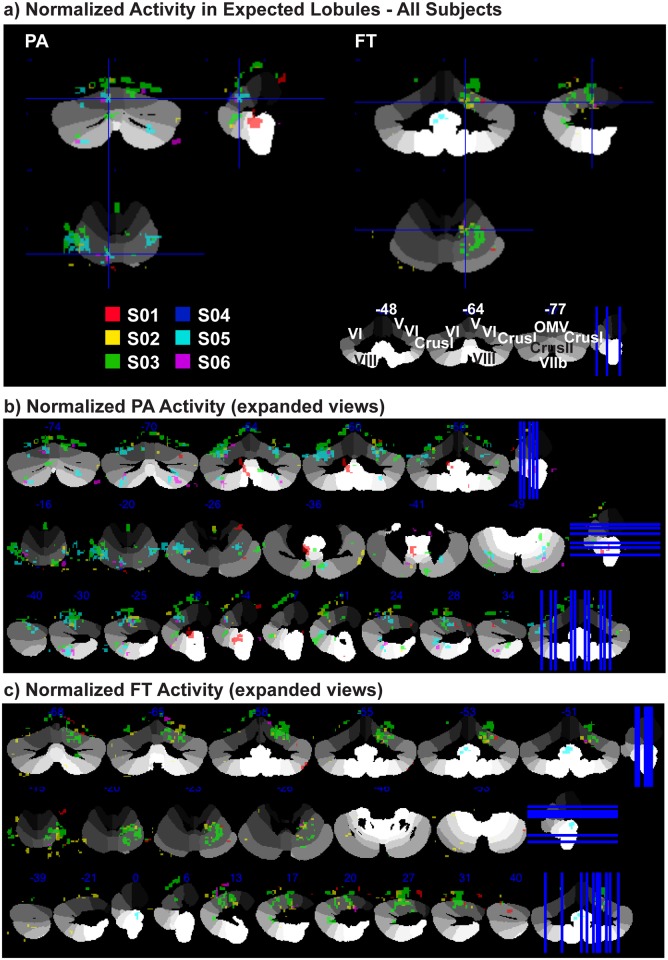
Inter-Subject Variability of Normalized Cb Activations. **a)** *left—*Normalized PA-activity, crosshairs at left paravermis VI; *right—*normalized FT-activity, crosshairs at ipsilateral (right) V; data are from all subjects, each subject is color-coded. **b)** PA activity is localized to oculomotor vermis (VIc and VII), bilateral *h*VI, and (right) *h*CrusI/II for all subjects. **c)** FT-activity in ipsilateral *h*V/VI for all subjects and ventral activity only for S01, S02 and S05. Activations from both tasks are clustered within the expected lobules yet inter-subject activations rarely overlap. Activations are T-maps from SUIT normalized functional images with Cb mask overlaid on SUIT template Cb [20]. Each subject is represented by a single color cluster-thresholded at *p* < .05 (AT).

The activation loci and T values for activations in each lobule of interest (in native/non-normalized space) are visible on the top of each panel and just below the axial slice, respectively, for each subject (Figs 2–6). Despite moving each individual brain into a common space (AC to [0 0 0], dorsal vermis in axial plane with orbitofrontal cortex), due to the structural discrepancies between individual Cb lobules, it is still difficult to infer how variable individual activations actually are. Though specifying individual variability of subject-specific functional zones is helpful for most clinical assessments and some experimental paradigms such as the present one, viewing individual variability in a common space may also be of interest. Fig 8 shows the T maps created from the same individual GLM from each subject run with (SUIT) normalized and masked functional images overlaid on the SUIT atlas; which is shaded in gray scale by lobule to assist with orientation (guide at bottom right of Fig 8a). Each subject is displayed in a different color and T-maps were thresholded as in earlier figures (AT through T = 8.5), based on brightness (darker—brighter). In Fig 8a, crosshairs are located at the same ROIs, left paravermis VI for the PA-related activity displayed in Fig 3 (Fig 8a *left*) and right *h*V for FT-related activity displayed in Fig 4 (Fig 8a *right*). Select cross sections of Cb are shown in 8b and 8c to display the variable location of activity between individuals within these lobules.

It is clear that there is a trend for PA-activity present in distinct bilateral clumps along *h*VI (axial slice of Fig 8a, left, and the leftmost axial slices in Fig 8b) and in *h*VIIb, and *v*VI-VIIb (including OMV—central sagittal slices in Fig 8b). FT-activity is generally confined to ipsilateral (R) *h*V-VI and *h*VIII (coronal slices in Fig 8a, right, and Fig 8c). Despite the fact that these activations normalize to the expected lobules, some subjects had additional activations in other motor and/or eye-movement lobules (i.e. *h*IX and *h*X, contralateral *h*V-VI). In addition, some activity was normalized outside of Cb, and inter-subject activations infrequently overlap within a single lobule. The remarkable differences in individual functional organization remain in both individual and common MNI space analyses. For example S01 and S06 infrequently overlap with any other subject, while S03 and S05 show a lot of PA overlap and S02 and S03 show a lot of FT overlap with each other in common space (Fig 8). Neither second level analysis from the six individual AT-thresholded nor from un-thresholded maps yielded significant activations for either task, even at the uncorrected level, and are therefore not shown.

## Discussion

### High-Resolution Structural and Functional Images from Multiple Systems in One Hour or Less

In this work we demonstrate the efficacy of combining high-density multi-element surface coils, two-channel transmission, and three-dimensional sensitivity encoded (3D SENSE) acquisitions to image subsystem-related activity in Cb in individual subjects. We were able to clearly differentiate activity throughout the entire Cb cortex including D in the DCN in a single session lasting less than one hour per subject. BOLD signal changes during motor activity resulted in concentrated clusters of activation observed within the expected lobules for each task in each subject: OMV and *h*CrusI/II for PA and ipsilateral *h*IV-VI for FT. In addition to these findings, neighboring activity in *h*VIIb/*h*VIII was consistently separable for the PA/FT tasks, respectively. Furthermore, depression of D activity related to computations involving more cognitive planning of eye movements (PA) was located caudal to that related to motor (FT) activity (Fig 7) as seen previously in both monkey and human studies [6,64,65]. To date, there have been no reports of such comprehensive high-resolution information obtainable in such a short time. Taken together with the uniformity of tSNR, especially for ventral and lateral Cb, these results represent positive improvements in Cb data acquisition.

Patients with Cb disorders often show abnormalities in eye movements and finger tapping (and/or other motor dysfunctions) [16,24], behaviors that can be readily elucidated on a case-by-case basis for clinical assessments using the above methods. This method eliminates the need to average across multiple patients with the same or very similar atrophy in order to associate functional impairments with specific regions and allows for pinpointing of structural and functional abnormalities at the level of the folium. This is also beneficial for patient-specific pre-surgical assessment of cerebellar infarcts related to vertebrobasilar stroke or arterial anastomoses [36,37], tumor resection [38], or for individual lesion-symptom mapping [39,40]. The ability to obtain structural and functional images quickly at high spatial resolution, enabling localization of activations specific to individual anatomy such as folia, is also critical when investigating highly specific physiological mechanisms such as patches of task-related activity and will prove helpful in further delineating the organizational separation of neural processes in human Cb. Therefore, the advantages of this technique apply to both research and clinical studies.

### Task-Specific Signal Changes Cluster Differently among Subjects

The current results support the topographical differentiation of functional motor planning within Cb; confirming patches of activations in subsystem-specific lobules involved in eye- and finger-movements. We were able to observe clearly differentiated clusters of activity specific to each motor subsystem in each subject: *h*VI and *h*CrusII/*h*VIIb activations during PA are consistently distinct from ipsilateral *h*V-VI and *h*VIII activations during FT (Figs 3, 4 and 6). Most of the observed activations were confined to the gray matter of the folia and loci of peak activation were aligned along one or two folium; voxels surviving FDR correction are generally confined to one row of grey matter, most easily seen as the brighter colors in the sagittal panels. This can be seen particularly clearly for all sagittal panels in Figs 2 and 4 (central panels), where activity from each task is confined between the white matter spines radiating out from deep Cb, and axial (rightmost) panels in Figs 3 and 4 where peak activity follows the curvature of these spines.

While the clarity of task differentiation is consistent across subjects, the relative arrangement of active zones are not identical. The images in Figs 2–7 demonstrate not only the gross difference in anatomical arrangement of Cb from person to person, but also the difference in the arrangement of these activations within that anatomy, and this inconsistency holds in normalized space (Fig 8). With increased CNR, anatomical areas with individual differences in functional processes can be elucidated and explored relative to a single subject [15,18]. For example, it has been shown (also using surface coils) that individual finger representations maintaining hand-based topography can be separated in Cb *h*V and *h*VIII, and differ between individuals [18]. It is likely that detecting these individualized patches is commonly hindered due to analyses that average brain activity over a group of subjects who’s activation patches do not overlap, or where inhomogeneity of the B1 field or reduced variation of the received signals provides insufficient CNR. When evaluating function pre-surgery one should aim to avoid both false-negatives and false-positives; and as seen when comparing the surface to the head coils (with individual ATs) major loci are present with both coils and active loci are more succinct with the surface coils. The larger between-subject variation in the AT-corrected values, as compared to the relatively stagnant SPMT-corrected values, (compare color bars, and see S1 Table) is another obvious precaution that should be further addressed in analyses of functional signals at the individual level for accurate localization of function. Much of the data presented here would not be visible with a standard voxel-wise FDR correction (i.e. SPMT) that does not take into account between-subject variations in the noise signal. Although many of the signal improvements seen here can be attributed to the high density of the coils themselves, perhaps some of the benefits arise from the additional fact that all 32 elements (as well as Cb) can be placed further within the volume transmit coil.

### Implications of Inter-Subject Variability on Group Analyses

The decreased acquisition time of the current experiment allowed us to show that a topographical differentiation of eyes and hands (two separate systems) is also possible within *h*VI and *h*VIIb/VIII, similar to separation of hand and foot movement seen by [4]; though the results here are less diffuse and were acquired much more quickly. However, Fig 8 shows that there is very little between-subject overlap of peak activations from the same task in normalized space (i.e. lobule VI in Fig 8a and 8b), and as can be expected the individual variations in the location of these small active zones lead to a loss of power at the group level resulting in no remaining active clusters when regressing the normalized and thresholded T-maps. Not to mention that the folium-specificity of the activations, as well as differentiating Cb activity from visual cortex activity is compromised or nullified in normalized space. In addition, if the normalized activations from *both* tasks were overlaid together for all subjects, the task-specific differentiations which are plainly visible within-subjects would become too jumbled to be interpreted as separable between-subjects.

It is possible that inter-subject differences in Cb physiology and/or arrangement of functional patches underlie the lack of overlap in normalized space; or this could be a result of the normalization method. Current normalization schemes [66,67] that fit brains into common space are standard procedure for functional image analyses at lower field strength and lower resolution. These are neither able to capture nor match high-resolution anatomical detail of individual subject data (see the folium-specific activations in Figs 2–7). Hence, to obtain meaningful functional overlap between subjects in common anatomical space and thus more power at group level analyses, smoothing of images on a scale larger than individual Cb folia is required, obscuring or even nullifying the variation in individual structural or anatomical architecture found at a higher resolution, and possibly artificially shifting the strength or location of task-related functional activations [68]. Group or cohort studies can maintain high-resolution by measuring signal changes within individualized functional-ROIs (task-related ROIs) and running statistics on extracted values in place of current techniques of normalizing images to a common space for voxel- or coordinate-based second level analyses. As investigations at higher-field strengths proliferate, new methods are being developed such as cross-normalization with 3T and 7T images [69] and subject-specific normalization utilizing high-resolution EPI images [35], both of which can allow for closer investigation of activations in Cb cortex by inflating and flattening the surface.

### PA Activity in CrusI/II: (Visual) Attention, or Cognition?

The clustered PA-related activations are located in five bilateral patches along lobule VI (L*h*, L paravermis, vermis, R paravermis, R*h*) and are arguably most significant outside of OMV; visible in the axial slices of Fig 3, most clearly for P01. Previous studies targeting visual perception and Cb have shown similar bilateral clustering throughout *h*VI in response to attending a moving grating [32], in medial Cb VI (vermis and paravermis) and *h*CrusI in response to saccadic errors [60], in bilateral *h*V-VI, OMV, and throughout CrusI/II in response to attended flashed visual stimuli [59], and in CrusI/II for complex motor tasks [65]. Both PA and FT used the same visual stimuli with moving dots cuing the motor activity, yet subsystem-specific activity was consistently separable throughout Cb as either eye- *or* finger-movement related, suggesting that observed patches of activity are related to motor planning and output rather than visual perception or attention alone since subjects were also attending these stimuli during the FT task. However, the location of these dots was only relevant during the PA task. It is therefore possible that the observed PA-related clusters, acting alone or in concert with CrusI/II, represent either possible task-relevant attention, target locations (and the return saccade, back to fixation) or target awareness, or a mapping of visual space in order to monitor and/or update performance on a visual task. Although the presence of vector mapping of saccades within Cb is currently unknown, spatial saccade maps are known to exist in superior colliculus and frontal eye fields [70,71].

The involvement of Cb in cognitive brain processes is a recently up-and-coming topic in cerebellar research [10,72,73]. The evolutionary increase in the size of CrusI and CrusII in humans and higher primates, as well as the anatomical and functional connections linking these lobules with the prefrontal cortex [72–76] has supported the idea that these areas also play some role in non-motor-related cognition and/or executive function. There has been much difficulty coming to agreement on which lobules of Cb are involved in which aspects of cognitive processing, possibly due to the observed individual variation in the arrangement of functional patches within a defined lobule (Figs 5 and 8) and/or the difficulty in differentiating attention from cognition in various experimental tasks. Topographical eye representations classically lie along lobule VI [5,9,10] and despite the presence of salient visual inputs there is no CrusI or CrusII activity during FT. With regard to the lucidity of these less classically defined cognitive signals, individual PA-related activations in CrusI and II were consistently comparable in strength to, if not stronger than, the FT-related activations in lobules V-VI. The lateral activity seen here could also be attributed to a cognitive component of executing an anti-saccade and/or suppression of a reflexive pro-saccade. Though the design of the PA task did not enable separation of signals related to pro-saccades (non-cognitive, reflexive eye movements) from those related to anti-saccades (more cognitive volitional eye movements), ventral activations (VIIIb) have previously been linked to anti-saccades in a group study [77]. However, neither these nor attentional mechanisms can be mutually excluded from those required to solve cognitive problems.

### Speculation on Mechanisms of Individual Variability of Functional Patches and Interpretation of BOLD Activations

Despite the fact that both tasks in the current experiment were performed in a blocked manner, it is surprising that T values for FT were often less than T values for PA, most notably in ventral Cb, since finger and hand movements are classically used more frequently to activate ipsilateral Cb ([18,46,48,49]—though acquisition times were at least three times longer in those experiments). The instructions for the FT task were simply “tap your thumb up and down two times per second”. This resulted in some subjects tapping on the bore of the scanner or on their own hand, adding somatosensory stimulation of the thumb (and hand) to the digit motion, while others only moved their thumb (motor-related activity, with no somatosensory input). These differing somatosensory inputs can directly relate to sensory-driven mossy fiber activity restricted by functional patches of PC activity, and can result in individual variability of the strength and spread of FT-related activity.

Task-related functional patches of activity are only inferentially relatable to cellular signaling due to the slow temporal resolution of our fMRI sequence and the limited number of studies investigating the behavior of Cb neurovasculature in response to cellular activity [78,79]. It could be argued that the arrangement of clustered folial activity observed here resembles previously observed neurophysiological recordings showing microbands, beams, colonies, or patches [80–83]. These types of zones have been related to subsystem-specific co-activation of localized clusters of PCs which topographically project to DCN and on to prefrontal and motor areas of the cerebrum (see [84] for review) and the activation of PCs can inhibit blood flow to and activation of neighboring PCs [85]. Taking into account existing literature, we may assume that the pre-defined clusters of neurons are indeed involved in topographic motor planning and that these clusters may effectively re-direct blood flow to patches of task-related activity, revealing the observed patches of BOLD signal change.

As previously mentioned, direct neuronal connections project activity from the PC layer in Cb cortex to the DCN, maintaining topographic functional organization [6,84], which output from Cb to the thalamus and other brain areas [64,86]. BOLD signal changes in D (Fig 7) were consistent with the present understanding [65] that motor connections are located more rostrally, where we see the most significant FT-related decrease in BOLD, while more complex and cognitive connections are located more caudally, where we observe a PA-related decrease. Activity from finger tapping in D was not as strong as that from eye movements; if the strength of cortical activity correlates to the strength of nuclear activity it is not surprising that the anticorrelation of FT activity was not as strong as PA anticorrelations in D (Fig 7, FT, purple circle), as this was the case for task-related cortical activity of this subject. The dorsal anticorrelation at -60 may reflect cortical activations related to hand position during FT, as the interposed nucleus is dorsal to the dentate and responds to proprioceptive limb positions; however, the dorsal deactivation occurs contralateral to the cortical activation. No changes in activity were observed in the fastigial nucleus during PA, which is known to be involved in eye movements. Though D is the largest and most thoroughly studied nucleus, no activations here survived cluster thresholding and we suggest that even higher resolution imaging may be able to clarify the separation of activations in DCN. The discrepancies in observed versus expected changes in signals from the DCN reflect the difficulty of inferring neuronal processes from functional data obtained by measuring the indirect consequence of changes in blood flow resulting from activity of many different types of Cb neurons [78,87]. It has recently been suggested that there is a skewed distribution of synaptic signaling weights, where only a subset of neurons within a functional network may drive the network independent of, for instance, the firing rate of most other neurons in the network [87]. These findings further complicate the interpretation of how neuronal signaling might be affecting blood flow.

## Conclusions

Pairing 32 channels of surface coils with excitation pulses steered with dual amplifiers facilitated imaging of the entire Cb, including the DCN (>5cm into the skull), despite the fact that these high-density multi-element coils only cover a part of the head. Since signal CNR was sufficient to consistently differentiate eye-related from hand-related motor activity and to confirm suspected cognition-related activity (particularly in lateral Cb) in each subject scanned, we can conclude that these coils are beneficial for fast imaging of Cb. Furthermore, the use of localized shimming and 3D-EPI acquisitions successfully avoided image distortion, artifacts, and signal losses commonly observed around Cb at high field strength and the entire scanning session lasted less than one hour.

Combining this acquisition scheme with newer analysis methodologies can allow for a larger number of investigations within one individual in a single scan session while freeing up space within the transmit coil. Future studies targeting event-related perception and learning paradigms can utilize these methods, specifically in human and non-human primate Cb, to further elucidate the neurovascular interplay of superficial and deep Cb and the dynamics and organization of zones or patches in humans to close the gap of inference with regard to the affects of neuronal processes on BOLD signal changes.

## Supporting Information

S1 Fig **Methods: Coil Comparison** Signal quality of the surface coils has been previously defined for visual cortex [21] but not for Cb, therefore one subject completed the PA-task twice, and images were acquired once with two of their coils and again with a standard full-head 32-channel coil (Nova, USA). Scan parameters and image analyses were identical to the methods in the main text. **Figure: Coil Comparison—PA Functional Activations.** *Bottom*: OMV (*v*VIc and *v*VII, crosshairs), *Top*: *h*VI, and CrusII (circled in white on coronal panels from both ROIs on the head coil image) are activated during the PA task—activation are shown in red-to-yellow, *Left*: Activations observed using a 32-channel full head coil (Nova Scientific, USA) *Right*: Activations observed using two 16-channel surface coils with the same subject. CrusII activations are also observed with the head coils (See Fig 5 and S3 Fig for CrusII activation with surface coils), and all activations are more succinct with the surface coils. Slice locations (in non-normalized MNI space) are displayed at the top of each panel and T-values at the crosshairs are displayed at the bottom of the axial (bottom left) panels. Refer to Fig 1a for a guide to anatomical lobule definitions.
(TIF)

S2 Fig Importance of Correct Coil Placement. Example images from one subject where coils were placed dorsal to Cb. Although most activation is limited to dorsal Cb, some activity in ventral Cb is still observable. Images can be compared to activations from the other six subjects with correct coil placement. Slice locations (in non-normalized MNI space) are displayed at the top of each panel and T-values at the crosshairs are displayed at the bottom of the axial (bottom left) panels. Refer to Fig 1a for a guide to anatomical lobule definitions.
(TIF)

S3 Fig Signal Strength and Activation Loci. T maps (Funct) and tSNR from a single PA run are overlaid on the T1w structural scan from one subject and aligned with the T2*w (T2*) structural images for six Cb ROIs (L and R paravermis VI, OMV, L paravermis VIIb, and R *h*VIIb and R *h*CrusII), indicated by crosshairs. It is clear that significant changes in BOLD signal are not restricted to areas with higher tSNR (yellow-white regions of tSNR images) nor do they occur spuriously around Cb vasculature (dark dots in the T2*w images). Slice locations (in non-normalized MNI space) are displayed at the top of each Funct panel and coordinates are the same for all three images. T-values and tSNR strength at the crosshairs are displayed at the bottom of the axial (rightmost) panels. Refer to Fig 1a for a guide to anatomical lobule definitions.
(TIF)

S1 Table Adaptive Cluster-Based (AT) versus SPM Voxel-Based (SPMT) FDR-Corrected Thresholds. NM—Normalised and Smoothed. *Thumb was tapped during the ENTIRE session (no rest period), so FT was not compared between head and surface coils.
(PDF)
