# Supplementary figures and images for: Single Session Imaging of Cerebellum at 7 Tesla: Obtaining Structure and Function of Multiple Motor Subsystems in Individual Subjects
Source: PLoS One. 2015 Aug 10;10(8):e0134933. doi: 10.1371/journal.pone.0134933 (PMC4530960; doi:10.1371/journal.pone.0134933)

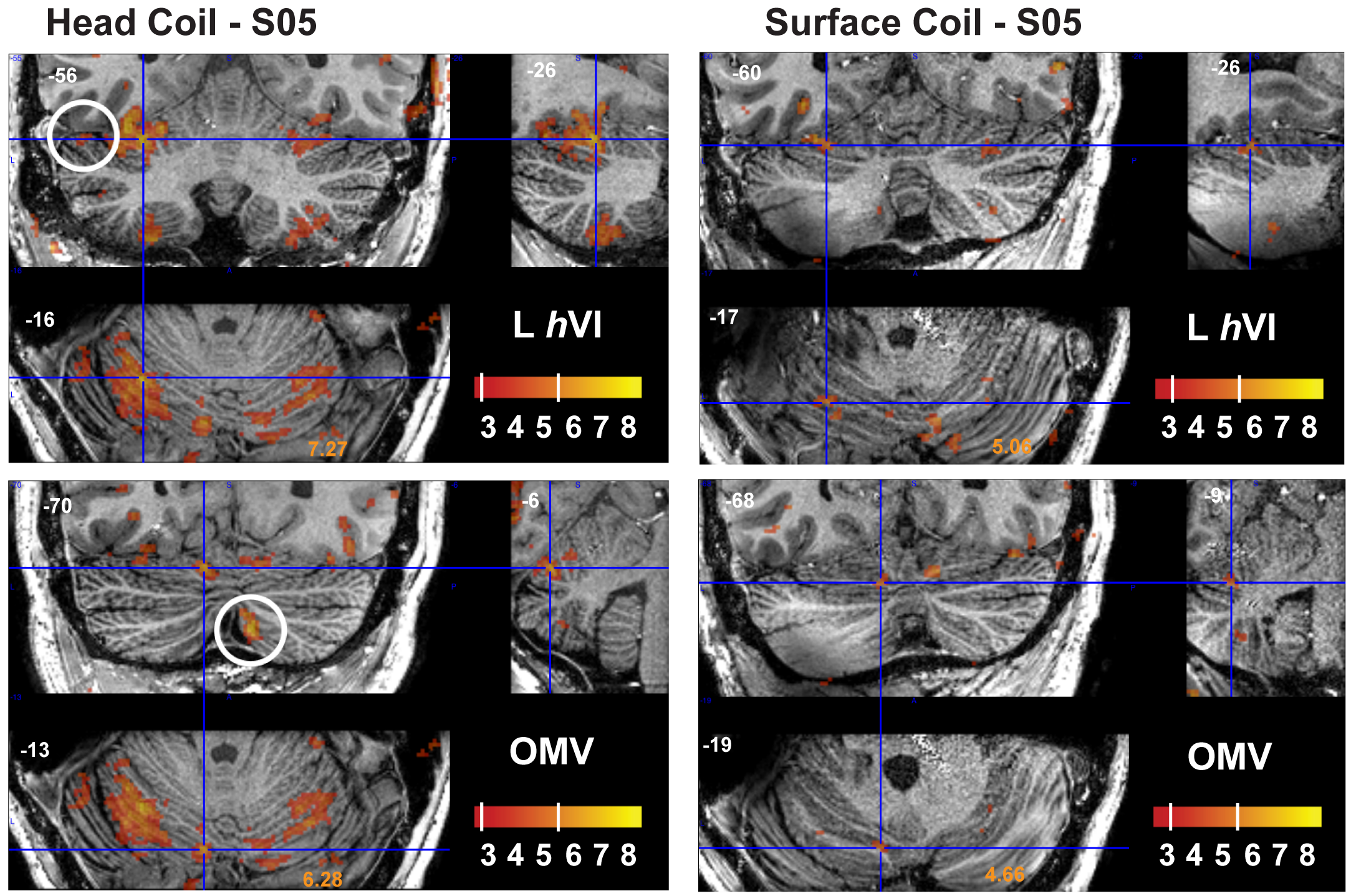

Supplement: S1 Fig — Methods: Coil Comparison Signal quality of the surface coils has been previously defined for visual cortex [21] but not for Cb, therefore one subject completed the PA-task twice, and images were acquired once with two of their coils and again with a standard full-head 32-channel coil (Nova, USA). Scan parameters and image analyses were identical to the methods in the main text. Figure: Coil Comparison—PA Functional Activations. Bottom: OMV (vVIc and vVII, crosshairs), Top: hVI, and CrusII (circled in white on coronal panels from both ROIs on the head coil image) are activated during the PA task—activation are shown in red-to-yellow, Left: Activations observed using a 32-channel full head coil (Nova Scientific, USA) Right: Activations observed using two 16-channel surface coils with the same subject. CrusII activations are also observed with the head coils (See Fig 5 and S3 Fig for CrusII activation with surface coils), and all activations are more succinct with the surface coils. Slice locations (in non-normalized MNI space) are displayed at the top of each panel and T-values at the crosshairs are displayed at the bottom of the axial (bottom left) panels. Refer to Fig 1a for a guide to anatomical lobule definitions. (TIF) [file pone.0134933.s001.tif]

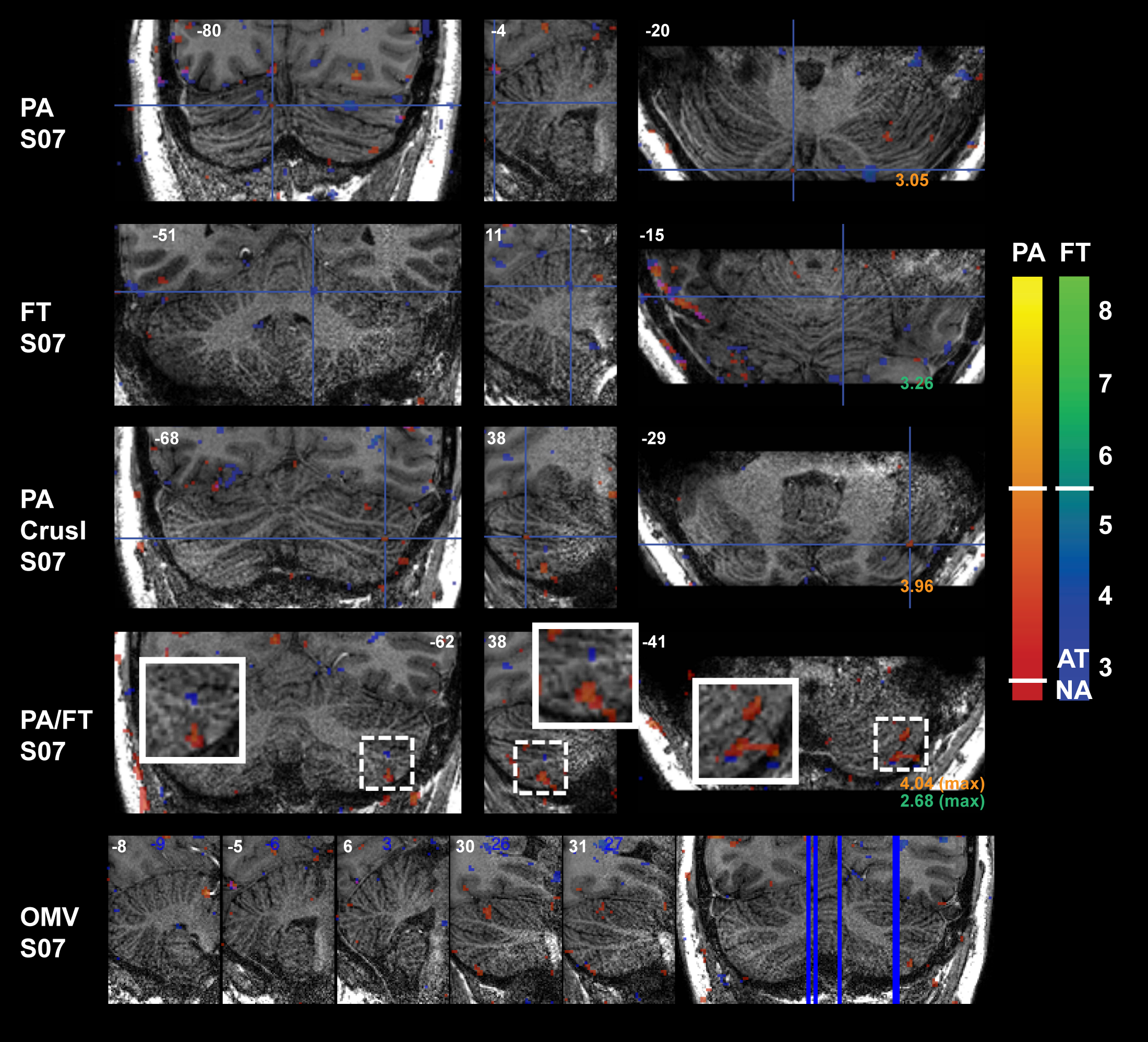

Supplement: S2 Fig — Example images from one subject where coils were placed dorsal to Cb. Although most activation is limited to dorsal Cb, some activity in ventral Cb is still observable. Images can be compared to activations from the other six subjects with correct coil placement. Slice locations (in non-normalized MNI space) are displayed at the top of each panel and T-values at the crosshairs are displayed at the bottom of the axial (bottom left) panels. Refer to Fig 1a for a guide to anatomical lobule definitions. (TIF) [file pone.0134933.s002.tif]

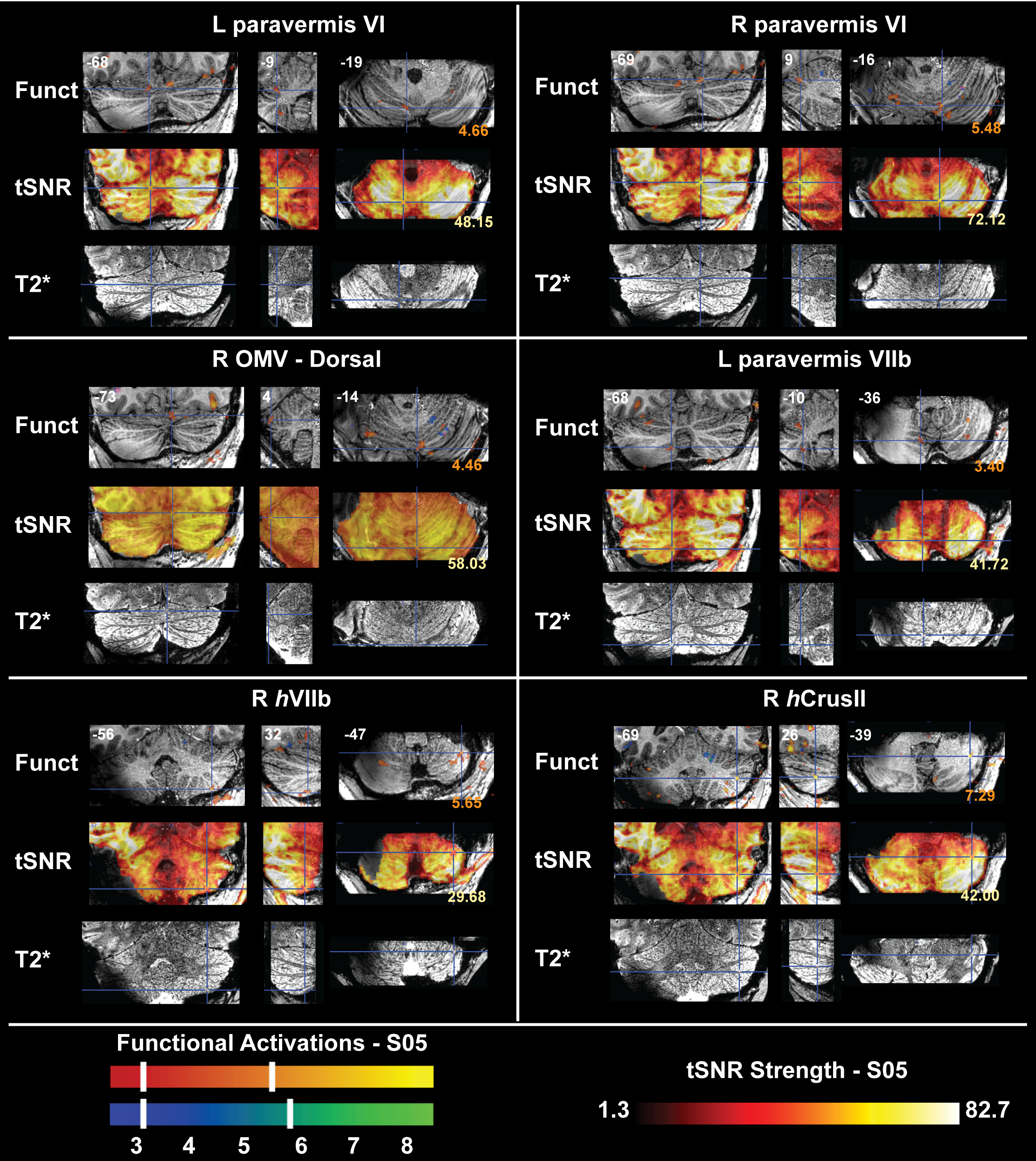

Supplement: S3 Fig — T maps (Funct) and tSNR from a single PA run are overlaid on the T1w structural scan from one subject and aligned with the T2*w (T2*) structural images for six Cb ROIs (L and R paravermis VI, OMV, L paravermis VIIb, and R hVIIb and R hCrusII), indicated by crosshairs. It is clear that significant changes in BOLD signal are not restricted to areas with higher tSNR (yellow-white regions of tSNR images) nor do they occur spuriously around Cb vasculature (dark dots in the T2*w images). Slice locations (in non-normalized MNI space) are displayed at the top of each Funct panel and coordinates are the same for all three images. T-values and tSNR strength at the crosshairs are displayed at the bottom of the axial (rightmost) panels. Refer to Fig 1a for a guide to anatomical lobule definitions. (TIF) [file pone.0134933.s003.tif]
