# Supplementary material for: Single Session Imaging of Cerebellum at 7 Tesla: Obtaining Structure and Function of Multiple Motor Subsystems in Individual Subjects
Source: PLoS One. 2015 Aug 10;10(8):e0134933. doi: 10.1371/journal.pone.0134933 (PMC4530960; doi:10.1371/journal.pone.0134933)
Supplement: S1 Table — NM—Normalised and Smoothed. *Thumb was tapped during the ENTIRE session (no rest period), so FT was not compared between head and surface coils. (PDF) [file pone.0134933.s004.pdf]

	Pro- versus Anti-Saccades (PA)		
	Individual AT	Individual SPMT	NM AT
S01	-3.1429 > T > 2.5904	+/- 5.4639	-3.1167 > T > 3.4231
S02	-3.4712 > T > 3.7725	+/- 5.4526	-4.3929 > T > 6.0207
S03	-3.8611 > T > 3.4088	+/- 5.4663	-3.2799 > T > 3.3436
S04	-3.3081 > T > 2.9102	+/- 5.4745	-3.4342 > T > 3.6921
S05	-3.4524 > T > 3.1405	+/- 5.4666	-2.8138 > T > 2.6274
S05 (head)	-4.0821 > T > 2.8409	+/- 5.4683	-3.4788 > T > 3.1093
S06	-3.2053 > T > 3.2128	+/- 5.4574	-2.5708 > T > 3.0297
S07	T > 2.8495	+/- 5.4605	-2.6720 > T > 3.8937
	Finger Tapping (FT)		
	Individual AT	Individual SPMT	NM AT
S01	-3.1252 > T > 2.9928	+/- 5.4632	T > 3.682
S02	-3.6386 > T > 3.9201	+/- 5.4517	-2.6404 > T > 4.8500
S03	-3.1687 > T > 2.7985	+/- 5.4587	-3.3849 > T > 2.7447
S04	-3.2823 > T > 2.8444	+/- 5.4644	--
S05	-3.8687 > T > 3.1489	+/- 5.7991	T > 2.4075
S05 (head)	T > 3.9093*	+/- 5.4679*	-1.8802 > T > 2.5988*
S06	-3.2650 > T > 2.7914	+/- 5.4581	-3.3780 > T > 3.0243
S07	--	+/- 5.4628	T > 2.5909
